# *In Vitro* Implantation Model Using Human Endometrial SUSD2^+^

**DOI:** 10.22074/cellj.2021.6979

**Published:** 2021-05-26

**Authors:** Marzieh Rahimipour, Mina Jafarabadi, Mojdeh Salehnia

**Affiliations:** 1. Department of Anatomy, Faculty of Medical Sciences, Tarbiat Modares University, Tehran, Iran; 2. Reproductive Health Research Centre, Tehran University of Medical Sciences, Tehran, Iran

**Keywords:** Embryo Implantation, Endometrium, Epithelial Differentiation, Mesenchymal Stem Cells, SUSD2+Cells

## Abstract

**Objective:**

This study evaluated a novel *in vitro* implantation model using human endometrial mesenchymal stem cells (EMSCs), SUSD2^+^, and myometrial smooth muscle cells (SMCs) that were co-cultured with mouse blastocysts as the surrogate embryo.

**Materials and Methods:**

In this experimental study, SUSD2^+^ MSCs were isolated from human endometrial cell suspensions (ECS) at the fourth passage by magnetic-activated cell sorting. The ECS and SUSD2^+^ cells were separately co-cultured with human myometrial muscle cells for five days. After collection of mouse blastocysts, the embryos were placed on top of the co-cultured cells for 48 hours. The interaction between the embryo and the cultured cells was assessed morphologically at the histological and ultrastructural levels, and by expression profiles of genes related to implantation.

**Results:**

Photomicrographs showed that trophoblastic cells grew around the embryonic cells and attached to theECS and SUSD2^+^ cells. Ultrastructural observations revealed pinopode and microvilli-like structures on the surfaces of both the ECS and SUSD2^+^ cells. Morphologically, the embryos developed to the egg-cylinder stage in both groups. Gene expression analysis showed no significant differences between the two groups in the presence of an embryo, but an increased expression of αV was detected in SUSD2^+^ cells compared to ECS cells in the absence of an embryo.

**Conclusion:**

This study showed that SUSD2^+^ cells co-cultured with SMCs could interact with mouse embryos. The co-cultured cells could potentially be used as an implantation model.

## Introduction

Implantation results from successful interactions between the embryo and endometrial epithelium during the mid-secretory phase of the menstrual cycle when the endometrium is receptive. At this so-called “window of implantation”, ultrastructural alterations occur on the surface of endometrial epithelial cells and serve as important implantation markers of the receptive endometrium (1, 2).

Human implantation proceeds through three main stages: apposition, adhesion, and invasion. During the apposition stage, the blastocyst interacts with the apical surface of the luminal epithelium through two-way molecular communication. During the receptive phase, the luminal epithelial surface changes from a non-adhesive to adhesive surface, which results in the appearance of pinopodes and reduction of lateral junctional complexes. During attachment, the embryo initiates a physical connection with the apical surface of the endometrial epithelium; however, during invasion, the trophoblast cells penetrate between the epithelial cells, migrates to and invades the blood vessels (3).

Impairment of implantation is considered a major cause of human pregnancy loss and infertility in assisted reproductive technologies (ART) (4, 5). Improving ART outcomes and preventing early pregnancy loss requires a better understanding of the mechanisms of interactions between the embryo and the endometrium during the implantation process. Since the *in vivo* study of human embryo implantation is unethical and has limitations, and the results of studies performed in animal models are not always applicable in humans, *in vitro* implantation models using human cells provide an alternative approach (6).

*In vitro* implantation models are categorized into several types (6). One mainly focuses on the interaction between endometrial epithelial cells and the embryo to evaluate the early stages of implantation (6-8). In another group of implantation models, late stages of implantation are studied through two-dimensional culture of endometrial stromal cells with an embryo (6, 9). In more complex models, endometrial epithelial and stromal cells are co-cultured with an embryo in a three-dimensional culture system, allowing the study of both early and late stages of implantation (6, 10-12). Because of limited access to human embryos, a number of studies have used surrogate embryos in designing implantation models (6). Several have employed mouse blastocysts (13, 14), while most have used trophoblast spheroids derived from trophoblastic cell lines (15, 16).

The human endometrium is a dynamic tissue that undergoes cyclical shedding and regeneration during each reproductive cycle. The identification of rare populations of adult stem cells in both the stratum functionalis and basalis suggest that they may play a critical role in endometrial regenerative activities (17-19). Endometrial stem/progenitor cells have adult stem cell characteristics of clonogenicity, high proliferative potential and multilineage differentiation potential (17, 20). They comprise epithelial, mesenchymal, and endothelial stem/ progenitor cells. Endometrial mesenchymal stem cells (EMSCs) are located ina perivascular region, and include pericytes and perivascular cells (21). They are identified by specific markers, such as co-expression of CD146 and PDGF-Rβ and a single marker, SUSD2 (W5C5) (17, 18, 22-26).

EMSCs have the potential to differentiate into several cell types *in vitro* (18, 26); thus, they may have extensive applications in cell therapy, tissue reconstruction, and regenerative medicine (27, 28). There are limited reports regarding the differentiation of endometrial stem/progenitor cells into endometrial glands and epitheliaupon transplantation under the kidney capsules of immunodeficient mice (29). Recently, we showed that CD146^+^ cells isolated from human endometrium differentiated into endometrial epithelial-like cells during co-culture with myometrial smooth muscle cells (SMCs) (30). Campo et al. (31) demonstrated that transplantation of cultured human endometrial side population (SP) cells, which were comprised of stromal and epithelial cells, to a decellularised porcine uterus resulted in some recellularisation with human vimentin positive stromal cells and rare cytokeratin positive epithelial cells. Recently, López-Pérez et al. (28) reported that injection of a human endometrial SP under kidney capsules induced reformation of human endometrium, which was confirmed by the presence of typical endometrial markers. They concluded that these cells had the optimum capacity to regenerate endometrial-like tissue.

Despite the differentiation potential of adult stem cells to endometrial-like cells, and according to our knowledge, few studies have designed an *in vitro* implantation model by using these cell types. Thus, the aim of the present study was to evaluate a novel *in vitro* implantation model that mimics the *in vivo* condition by using human EMSCs co-cultured with human myometrial SMCs to assess implantation with mouse blastocysts as the surrogate embryo.

## Materials and Methods

All reagents were purchased from Sigma Aldrich (Germany) unless otherwise indicated.

### Human tissue collection

For this experimental study, human endometrial (n=10) and myometrial (n=10) tissues were obtained from healthy fertile women (aged 25-40 years) during the proliferative phase, and who were undergoing hysterectomies for non-pathological conditions. The women had not taken any exogenous hormones for three months before surgery (Table S1, See Supplementary Online Information at www.celljournal.org). Samples were transported to the laboratory in equilibrated and pre-warmed Leibovitz’sL-15 medium supplemented with 10 mg/ml human serum albumin, 100 IU/ml penicillin and 100 μg/ml streptomycin within 1-2 hours.

The Ethics Committee of the Medical Faculty of Tarbiat Modares University (Tehran, Iran, no.1394.137) approved this experimental study and written informed consent was received from all patients.

### Experimental design

Figure S1 (See Supplementary Online Information at www.celljournal.org) shows the experimental design. Human endometrial cells were isolated mechanically and enzymatically from endometrial tissues and cultured up to the fourth passage. Then, the SUSD2^+^ cells were sorted by magnetic activated cell sorting (MACS) and their characteristics were confirmed by immunohistochemistry. The endometrial cell suspensions (ECS) and sorted SUSD2^+^ cells were separately co-cultured with myometrial smooth muscle for five days, after which the cultivation period was extended for an extra 48 hours in the presence or absence of mouse blastocysts in order to establish two *in vitro* embryo implantation models. At the end of the culture periods, the endometrial (ECS and SUSD2^+^ cells) and embryonic cell interactions were assessed by morphological, ultrastructural and molecular studies.

### Morphological evaluations of endometrial and myometrial samples

Ten samples each of endometrial and myometrial tissue were separately fixed in Bouin’s solution, processed, embedded in paraffin wax and sectioned into 7 µm thicknesses. After hematoxyline and eosin (H&E) staining, the sections were observed with a light microscope and their normal morphology was evaluated (32).

### Isolation of human endometrial cells

Human endometrial cells were isolated from tissues as per the Chan et al. (33) method. Briefly, human endometrial tissue was washed in phosphate-buffered saline (PBS) and then cut into small 1×1 mm pieces within Dulbecco’s modified Eagle’s Medium/Hams F-12 (DMEM/F-12) that contained 100 mg/ml penicillin G sodium and 100 mg/ml streptomycin sulphate B. The tissue fragments were separated into single cells using collagenase type 1 (300 μg/ml) and deoxyribonuclease type I (40 μg/ ml) for 90 minutes together with a mechanical method. To eliminate glandular and epithelial components, the cell suspension was passed sequentially through sieves of mesh at sizes of 100 and 40 µm (SPL Life Sciences Co., Korea), respectively (34). Endometrial stromal cells in the supernatant were cultured using DMEM/F-12 that contained antibiotics and 10% fetal bovine serum (FBS, all from Invitrogen, UK) and incubated at 37˚C in 5% CO_2_ . The cells were cultured up to passaged when they reached to 80-100% confluency, used for the following assessments.

### Confirmation of endometrial mesenchymal cells using flow cytometry

A number of the passage-4 endometrial cells were evaluated for mesenchymal (CD90, CD73 and CD44) and hematopoietic markers (CD45 and CD34) by flow cytometric analysis. A total of 1×10^5^ endometrial cells were suspended in 50 μl of PBS and incubated with direct fluorescein isothiocyanate (FITC)-conjugated antibodies (anti-human CD90, CD44, and CD45, 1:50 dilutions) and direct phycoerythrin (PE)-conjugated antibodies (anti-human CD73 and CD34; 1:50 dilutions) at 4˚C for 45 minutes. Finally, 200 μl of PBS was added and the cells were examined with a FACSCaliburcytometer (Becton Dickinson, Germany). The flow cytometric analysis was repeated three times.

### SUSD2^+^ cell isolation by magnetic-activated cell sorting

After the fourth passage, the cultured human endometrial cells were washed, resuspended (up to 1×10^7^ cells/100 μl) in cold PBS and incubated with mouse anti-SUSD2 monoclonal antibody (327401, 8:200, Biolegend, UK) at 4˚C for 30 minutes. The cells were washed with MACS separation buffer (130-091-221, Miltenyi Biotec, Germany), then they were incubated with goat anti-mouse IgG Microbeads antibody (130047102, 20:100, Miltenyi Biotec, Germany) at 4˚C for 20 minutes. The cell suspensions were washed and run through the MACS column, followed by washing the column for three times with 500 μl MACS separation buffer. Magnetically labelled cells (SUSD2^+^) were mostly retained on the column and the unlabelled cells (SUSD2^-^) were eluted. Trypan blue staining (0.4%) was performed to determine SUSD2^+^ cell viability following MACS sorting. All experiments were repeated three times.

### Immunocytochemistry of sorted endometrial SUSD2^+^ cells

The purity of the magnetic bead-sorted human endometrial (SUSD2^+^) cells was assessed by immunocytochemistry (n=3 samples). These cells were incubated with mouse anti-SUSD2 monoclonal antibody (327401, 8:200, Biolegend, UK) at 4˚C for 30 minutes. After washing the cells with PBS, they were incubated with secondary goat anti-mouse polyclonal antibody conjugated with Alexa Fluor® 488 (405319, 1:100 in PBS, Biolegend, UK) for 2 hours at 37˚C and washed three times with PBS. Nuclei were counterstained with 4’, 6-diamidino-2-phenylindole (DAPI, D9542, Sigma, Germany) for 30 seconds. For negative controls, the cells were treated with the 10% unimmunized mouse serum in PBS instead of primary antibody. All experiments were repeated three times.

### *In vitro* culture of human myometrial cells

After dissection, the tissue fragments of the myometrium were cultured according to the explant method as reported by Fayazi et al. (30). Briefly, the human myometrial tissues (n=10) were washed with PBS and then cut into 1×1 mm pieces in DMEM/F-12 that contained 100 mg/ml penicillin G sodium and 100 mg/ml streptomycin sulphate B. Finally, the fragments were placed in each well and the emerging cells were allowed to grow in complete DMEM/F-12 supplemented with 10% FBS to confluency at 37˚C and 5% CO_2_ for three weeks. The medium was changed every two days. The characteristics of isolated myometrial cells were confirmed by immunocytochemical analysis. 

### Immunocytochemistry of myometrial cultured cells

Passage-2 trypsiniszed myometrial cells (n=3 samples) were cultured on cover slips. After attachment, the cultured cells were washed three times with PBS, fixed with 4% paraformaldehyde at 4˚C for 20 minutes, and permeabilised with 0.3% TritonX-100 for 45 minutes. Non-specific binding was blocked with 10% normal goat serum in PBS. Cells were separately incubated with the SMC markers, mouse anti-vimentin monoclonal antibody (V6389, 3:100 in PBS, Sigma-Aldrich, Germany) and rabbit anti-alpha smooth muscle actin polyclonal antibody (ab5694, 1:100 in PBS, Abcam, UK) at 4˚C overnight. The cells were washed in PBS three times, and incubated with secondary antibodies rabbit anti-mouse polyclonal antibody conjugated with Texas red (315-075-003, 3:100 in PBS, Biolegend, UK) and goat anti-rabbit IgG conjugated with FITC (ab6717, 1:1000 in PBS, Abcam, UK) at 37˚C for 2 hours. For negative controls, 10% unimmunized mouse serum in PBS was used instead of primary antibody. The immunocytochemistry analysis was repeated three times.

### Collection of mouse blastocysts

Adult, 8-10 week-old female (n=40) and 8-12 week-old male (n=10) National Medical Research Institute (NMRI) mice were housed and used under standard conditions for laboratory animals at Tarbiat Modares University (Iran). The Committee for Animal Research of the University approved all of the experimental procedures. Adult female mice were super ovulated with an intraperitoneal (i.p.) injection of 7.5 IU pregnant mare serum gonadotropin (PMSG, Folligon, Intervet, Australia) and then by an i.p. injection of 10 IU human chorionic gonadotropin hormone (hCG, Pregnyl, Netherlands) 48 hours later. After the second injection, the mice were individually mated with fertile males. On the morning of the fifth day of pregnancy, blastocysts were flushed from the uterine horns and the hatched blastocysts were used for the experiments.

### Implantation models using SUSD2^+^ cells and endometrial cell suspensions 

The SUSD2^+^ cells (group 1) and ECS (group 2) were separately co-cultured with myometrial cells as two experimental groups. In each group, 10^4^ SUSD2^+^ or ECS cells were cultured in 48-well plates with 5×10^3^ myometrial cells per well for five days. On the fifth day of culture, the mouse blastocysts were placed on the top of each well, with n=5 embryos in each well and a total of 45 embryos in each group for at least 9 repeats. The groups co-cultured in the absence of mouse blastocysts were considered to be the control groups. Then, these cells were cultured and monitored up to an additional 48 hours and evaluated morphologically by inverted microscope, live/dead staining, scanning electron microscope (SEM) and analysis of gene expressions related to implantation.

### Live/dead staining

We assessed the viability of the embryos and cells at 48 hours after the embryo culture on the top of each of the co-culture experimental groups by using a live/dead viabilitykit (L-3224, Invitrogen, UK). For this purpose, the cells were incubated with calcein AM (green) and ethidium homodimer-1 (EthD-1, red) for intracellular esteraseactivity and plasma membrane integrity, respectively, according to the manufacturer’s instructions. Then, the embryos and cells were observed under a fluorescent microscope (Nikon TE2000, Japan). This experiment was performed in triplicate.

### Scanning electron microscope

After two days of co-culture of the experimental groups with embryos, we examined the ultrastructure and interaction of the implanted embryos with co-cultured cells by SEM and compared them with their respective controls (groups without embryos). The specimens were fixed in 2.5% glutaraldehyde and post-fixed with 1% osmium tetroxide in PBS for two hours. After dehydration in an ascending ethanol series, the specimens were dried in a freeze dryer (Snijders Scientific LY5FME, Netherlands), mounted and coated with gold particles (BalTec, Switzerland) and examined under SEM (Philips XL30, Netherland). These experiments were repeated three times.

### Expression of implantation genes by real-time reverse transcription polymerase chain reaction

We evaluated the expressions of genes related to implantation: *αV* and *β3* integrin, interleukin-1 receptor (*IL-1R*), leukaemia inhibitory factor (*LIF*) and LIF receptor (*LIFR*). Total RNA was extracted from the collected cells after seven days of co-culture in both groups in the presence and absence of mouse embryos (5 embryosper well and, in total, 15 embryos per group with at least 3 replicates) using TRIzol (Invitrogen, UK). The concentration of isolated RNA was determined by a spectrophotometer, then cDNA was synthesized using a cDNA kit (Thermo Scientific, Lithuania, EU) in a total volume of 20 μl and the samples were stored at -80˚C until analysis. As shown in Table 1, the primers were designed based on human mRNA coding sequences using GenBank (http://www.ncbi.nlm.nih.gov) and synthesized at CinnaGen Company (Iran). The *β-actin* gene was used as an internal control.

**Table 1:** Characteristics of primers used for the real-time RT-PCR assay


Target gene	Primer pair sequences (5'-3')	Accession number	Fragment size (bp)	Temp. (˚C)

**αV**	F: ATCTCAGAGGTGGAAACAGGA	NM_002210.4	21	58.09
	R: TGGAGCATACTCAACAGTCTTTG		23	58.68
**β3**	F: AGTAACCTGCGGATTGGCTTC	NM_000212.2	21	60.68
	R: GTCACCTCGTCAGTTAGCGT		20	59.76
*L**IF*	F: CCAATGTGACGGACTTCCC	NM_002309.4	19	58.15
	R: TACACGACTATGCGGTACAGC		21	59.94
**LIFR**	F: TGTAACGACAGGGGTTCAGT	NM_001127671.1	20	58.58
	R: GAGTTGTGTTGTGGGTCACTAA		22	58.46
**IL-1R**	F: GGCACACCCTTATCCACCAT	NM_001261419.1	20	59.74
	R: GCGAAACCCACAGAGTTCTCA		21	60.54
**β-actin**	F: TCAGAGCAAGAGAGGCATCC	NM_001101.3	20	60.5
	R: GGTCATCTTCTCACGGTTGG		20	60.5


RT-PCR; Reverse transcription polymerase chain reaction.

After cDNA synthesis, real time reverse transcription polymerase chain reaction (RT-PCR) was performed by an Applied Biosystems real-time thermal cycler according to a QuantiTect SYBR Green RT-PCR kit (Applied Biosystems, UK). For each sample, the target genes and the reference gene were amplified in the same run and melting curve analysis was used to confirm the amplified product. Real-time thermal conditions included a holding step: 95˚C for 10 minutes and a cycling step: 95˚C 15 seconds and 60˚C 1 minute, followed by a melting curve step: 95˚C 15 seconds, 60˚C 1 minute and 95˚C 15 seconds. The Pfaffl method (35) was used to determine the relative quantification of target genes to the housekeeping gene. All experiments were repeated three times.

### Statistical analysis

Quantitative variables were expressed as mean ± SD. The results of real-time RT-PCR were compared by the independent samples t test, one-way ANOVA and post hoc Turkey’s tests. P≤0.05 were considered statistically significant. Statistical analysis was performed using SPSS software (V24, SPSS Inc., Chicago, IL, USA). 

## Results

### Morphology of human endometrial and myometrial tissue

**Fig.1:**
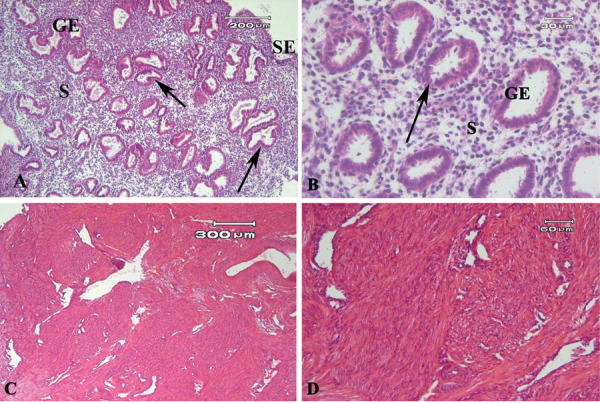
Light microscopic observation of hematoxyline and eosin (H&E) stained sections. **A, B.** Human endometrial tissue sections, **C, D.** Human myometrium tissue sections. GE; Glandular epithelium, SE; Surface epithelium, and S; Stroma, simple columnar epithelium of gland (black arrow).

H&E stained sections of human endometrial tissue from the proliferative phase showed typical morphologies of the basalis and functionalis layers (Fig .1A, B). The glands were lined with simple columnar epithelium (arrow) and the stroma comprised fibroblast-like stromal cells. The normal morphology of SMCs in myometrial tissue after H&E staining are presented in Figure 1C and D.

### The morphology of cultured endometrial cell suspensions, SUSD2^+^ and myometrial cells

**Fig.2:**
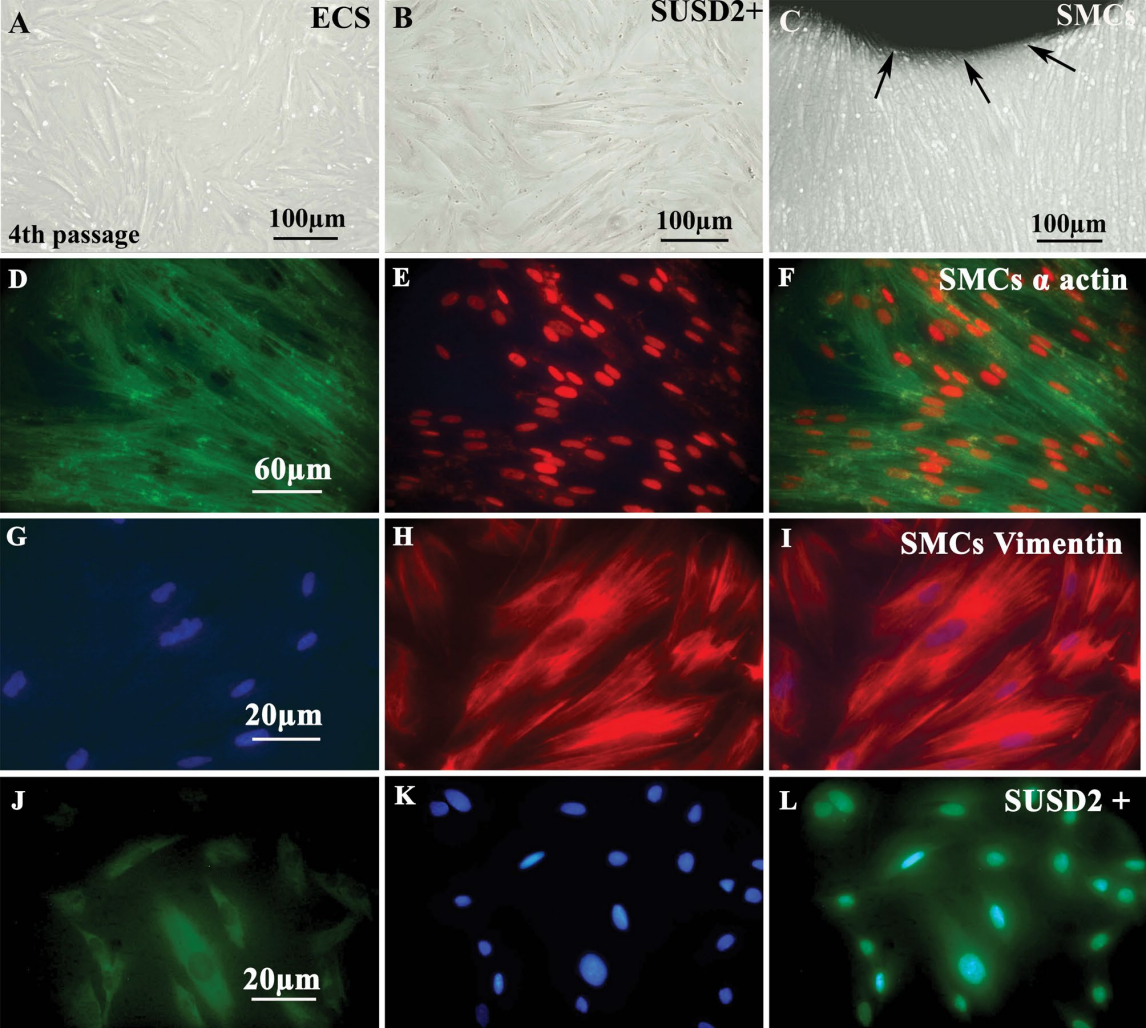
Phase contrast and immunohistochemistry of human cultured endometrial amd myometrial cells. **A.** Phase contrast imaging of cultured human endometrial cell suspension (ECS) at passage 4, **B.** SUSD2^+^ cells after separation and sorting by magnetic-activated cell sorting and **C.** Human myometrial cultured smooth muscle cells (SMCs) 25 days after tissue culture. The black arrows show the border of tissue explant as a dark colour. **D-F.** Immunofluorescence staining of cultured myometrial cells with anti-alpha SMC actin antibodies. **D.** The cytoplasm is stained green with fluoresce in isothiocyanate (FITC)-conjugated secondary antibody, **E.** The nucleus is stained red with propidium iodide, and **F.** The merged image is presented in third column. **G-I.** Immunofluorescence staining of cultured myometrial cells with anti-vimentin antibodies. **G.** The cytoplasm is stained red with Texas red-conjugated secondary antibody, **H.** The nucleus is stained blue with 4’, 6-diamidino-2-phenylindole (DAPI), and** I.** The merged image is presented in the third column. **J-L. **Immunocytochemistry of magnetic activated cell sorting (MACS)-sorted cells for SUSD2 is demonstrated. The sorted cells were shown in (J) that stained with nuclear staining by DAPI were demonstrated in (K) and the merged figure is shown in (L). The green colour shows the positive reaction for SUSD2 expression and blue colour is related to nuclear staining by DAPI.

Dissociation of the endometrial tissue yielded single cell suspensions of epithelial cells and stromal cells. At passage 4, cultured ECS showed a typical fibroblast morphology (Fig .2A). The morphology of cultured SUSD2-sorted cells under inverted microscope is shown in Figure 2B. Explant cultures of myometrium yielded stellate or triangular shaped cells (Fig .2C), which became confluent after three weeks of culturing. Their immunostaining with α-smooth muscle actin and vimentin are presented in Figure 2 D-F and G-I, respectively, which confirmed their smooth muscle identity. 

### Phenotypic analysis of cultured endometrial stromal cells

After the fourth passage, the endometrial cells showed the typical mesenchymal stem cell surface phenotype for markers CD73 (97.7 ± 1.5%), CD90 (87.3 ± 2.1%) and CD44 (69.1 ± 2%). They were negative for hematopoietic markers CD34 (1.99 ± 0.1%) and CD45 (1.03 ± 0.06%) as mentioned in our previous study (36).

### Cell survival and the percent of SUSD2^+^ cells after sorting

The survival rate of sorted SUSD2^+^ cells after MACS isolation was 91 ± 3.4%. The confirmation of the sorted cells by immunocytochemistry for the SUSD2 marker showed that 88 ± 2.7% of the nucleated cells were positive for the SUSD2 antibody (Fig .2J-L).

#### Light microscopic observation of implantation models

**Fig.3:**
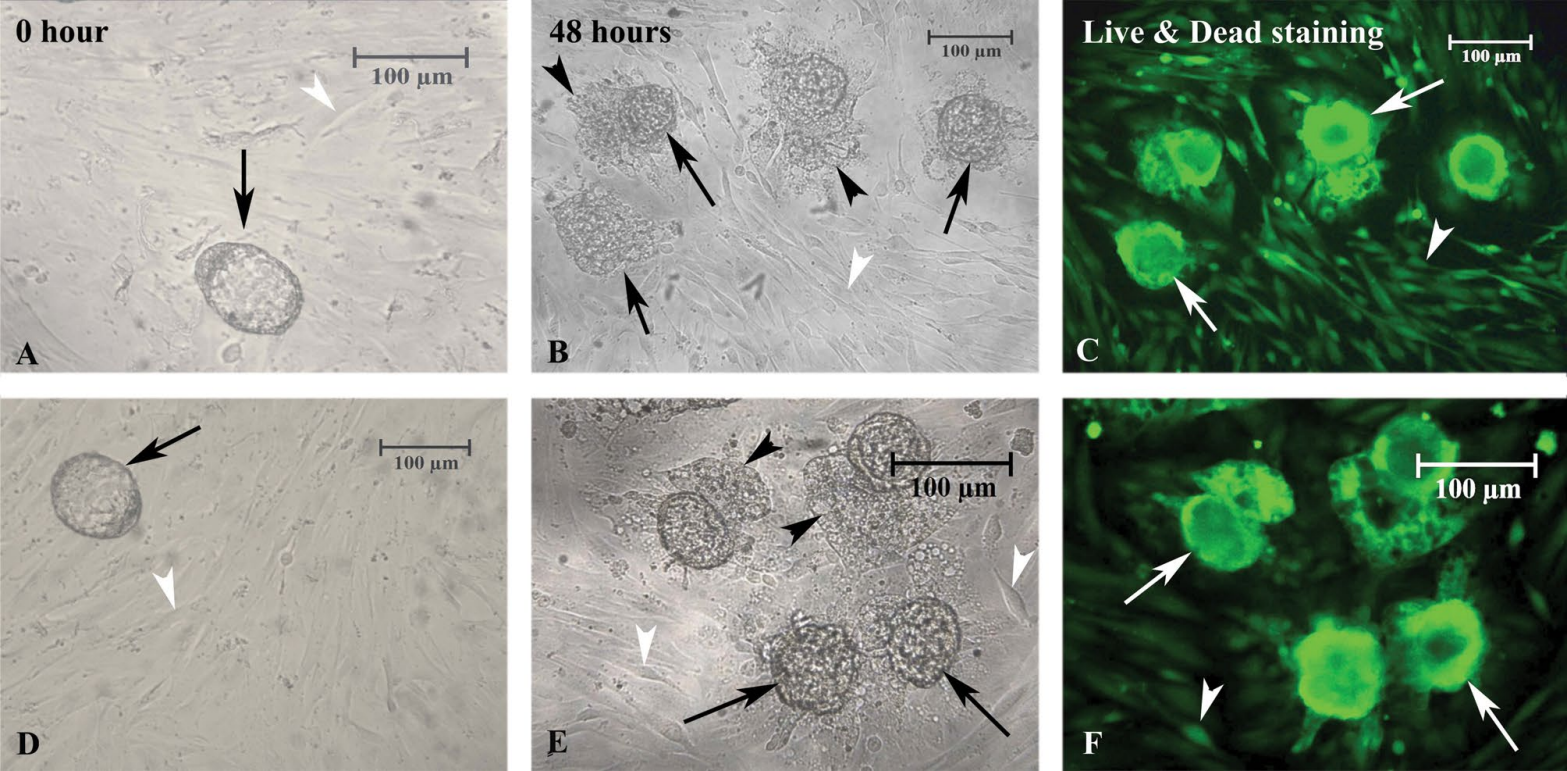
Phase contrast imaging of implantation models using mouse blastocyst in studied groups. **A-C.** The implantation of mouse embryo on top of the human endometrial cell suspension (ECS) and **D-F.** The embryo implanted on top of SUSD2^+^ cells co-cultured with myometrial smooth muscle cells (SMCs). First column showed the figures at the start (0 hours) of co-culture and in the second column showed after 48 hours of co-culture. The black arrows indicate the mouse blastocysts during the co-culture period, the black arrowheads indicate the expanded trophoblastic cells, and the white arrowheads indicate the human endometrial cells co-cultured with SMCs as the feeder layer. Fluorescence microscopy imaging of implanted mouse blastocyst in studied groups using a live/dead viability kit. **C.** ESC co-cultured with myometrial SMCs and** F.** The SUSD2^+^ cells co-cultured with myometrial SMCs. The white and black arrows indicate the mouse blastocysts and the white arrowheads demonstrate the feeder layer. Viable cells were stained green.

Phase contrast imaging of implantation models using mouse blastocyst in studied groups were demonstrated in Figure 3A-F. The morphology of ECS and SUSD2^+^ cells co-cultured with myometrial SMCs without embryos showed a flattened monolayer of spindle-shaped cells after the cultivation period (Fig .3, first column).

However, the implanted mouse embryos incubated with the co-cultures demonstrated similar morphological features between the ECS and SUSD2^+^ groups. The trophoblastic cells migrated from the embryos and proliferated, and the embryonic cells spread on the endometrial/myometrial cell layer and were tightly attached (Fig .3, second column).

The vital live/dead staining of the embryos on the co-cultured cells shows that all of the mouse implanted embryos were viable after 48 hours of culture (Fig .3, third column).

#### Electron microscopic observation of implantation models

**Fig.4:**
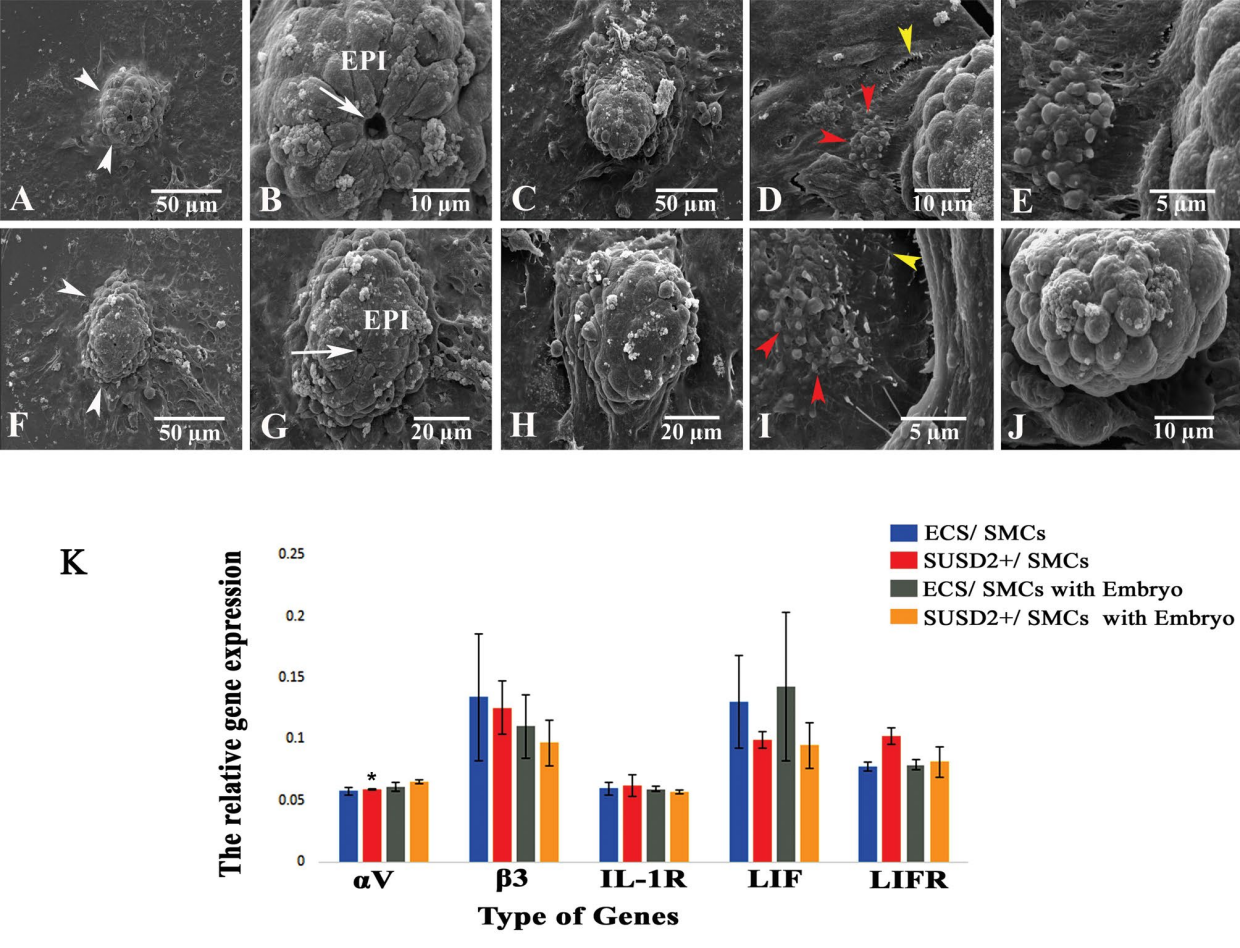
Scanning electron micrographs of studied implantation models. **A-E.** The micrographs of mouse blastocyst implantation on top of the endometrial cell suspension (ECS) co-cultured with myometrial smooth muscle cells (SMCs) with different magnifications, **F-J. **The figures of mouse embryo that implanted on the SUSD2^+^ cells co-cultured with myometrial SMCs at different magnifications. Two types of morphology were seen in the mouse embryos. White arrowheads; Mouse embryo (egg-cylinder), White arrow; Lumen of the pro-amniotic cavity, Red arrowheads; Pinopode-like structures, Yellow arrowheads; Microvilli-like structures, and EPI; Pluripotent epiblast, and **K.** Comparison of expression profiles of genes related to implantation relative to *β-actin* as the housekeeping gene are presented in ECS and SUSD2^+^ cells co-cultured with SMCs in the absence and in the presence of embryos. *; Significant differences with ECS/SMCs group (P=0.003), *IL-1R*; Interleukin-1 receptor, and *LIFR*; Leukaemia inhibitory factor receptor.

SEM evaluation of mouse blastocyst implantation on top of the ECS co-cultured with myometrial SMCs and the SUSD2^+^ cells co-cultured with myometrial SMCs are shown in Figure 4A-E and F-J, respectively. Ultrastructural evaluation of the human ECS or SUSD2^+^ cells co-cultured with human myometrial cells demonstrated that both had similar flattened spindle-shaped and flattened cells attached to the plate (Fig .4A, F). Some surface apical projections were seen on the endometrial cells adjacent to the implanted embryos, and these projections were similar to pinopodes (red arrowhead, Fig .4D, I) and microvilli (yellow arrowhead, Fig .4D, I).

The images obtained from the SEM indicated vertical growth of the embryos and the formation of mouse egg-cylinders in both studied groups. However, two different morphologies related to implanted embryos were observed at the ultrastructural level in each group: one with the presence of polarized cells (epiblast cells) arranged radially around the lumen of the pro-amniotic cavity and the other without polarized cells. This observation showed embryonic development on these co-cultures.

#### Molecular analysis of implantation models

Figure 4K shows a comparison of the ratios of gene expressions related to implantation (*αV, β3, IL-1R, LIF* and *LIFR*) to *β-actin* in both implantation models to the expression of *β-actin* in both implantation models in the absence or presence of embryos.

In the absence of embryos, the ratios of the expression of genes to that of the housekeeping gene were 0.65 ± 0.01 (*αV*), 0.97 ± 0.18 (*β3*), 0.57 ± 0.01 (*IL-1R*), 0.81 ± 0.11 (*LIF*) and 0.95 ± 0.18 (*LIFR*). These ratios in SUSD2^+^ cells co-cultured with SMCs were 0.59 ± 0.005 (*αV*), 1.25 ± 0.21 (*β3*), 0.62 ± 0.08 (*IL-1R*), 1.02 ± 0.07 (*LIF*) and 0.99 ± 0.06 (*LIFR*). The expression of *αV* significantly increased (P=0.003) in SUSD2^+^ cells compared to ECS. Expressions of the *β3, IL-1R, LIF* and *LIFR* genes were not significantly different between the two groups.

In SUSD2^+^ cells that were co-cultured with the embryo had the following ratios of expression: *αV* (0.61 ± 0.03), *β3* (1.10 ± 0.25), *IL-1R* (0.59 ± 0.02), *LIF* (0.79 ± 0.04) and *LIFR* (1.42 ± 0.60) compared to* β-actin*. In the ECS cells, these rates were: 0.57 ± 0.02 (*αV*), 1.34 ± 0.51 (*β3*), 0.59 ± 0.04 (*IL-1R*), 0.77 ± 0.04 (*LIF*) and 1.30 ± 0.37 (*LIFR*). There was no significant difference between the two groups.

The expression of genes related to implantation was not significantly different between the groups in the presence and absence of mouse embryos.

## Discussion

Considering the differentiation potential of EMSCs, SUSD2^+^ stem cells were used in the present study, for the first time, to create a new model of embryo implantation in comparison with an endometrial cell suspension that used mouse blastocysts as the surrogate embryo. For this purpose, SUSD2^+^ mesenchymal stem cells were isolated and co-cultured with SMCs and mouse blastocysts. Our results at the morphological and ultrastructural levels showed that the mouse blastocysts could interact with ECS and SUSD2^+^ cells and advance through the early stages of *in vitro* development within 48 hours. Moreover, electron micrographs indicated the ultrastructural changes in endometrial epithelial-like cells, including the appearance of pinopode-like and microvilli-like structures that are markers for early stages of implantation. 

In another point of view, the ultrastructure of mouse embryos in the present study indicated the progression of their developmental stages and the formation of an egg-cylinder. This stage of *in vitro* development is observed before gastrulation in mouse embryos (37, 38).

Evaluation of the expression of genes related to implantation in ECS and SUSD2^+^ cells after co-culture with SMCs indicated that these genes were expressed. Moreover, there was an increase in the expression of αV in SUSD2^+^ cells compared to ECS. No significant differences were observed in the expressions of the other genes (*β3, IL-1R, LIF* and *LIFR*) between these groups. These data showed that SUSD2^+^ EMSCs are multipotential cells that could differentiate to endometrial-like cells. Similarly, Fayazi et al. (30) revealed that CD146^+^ endometrial cells could express genes related to implantation, including secreted phosphoprotein 1 and matrix metalloproteinase-2, after differentiation into epithelial-like cells. In agreement, Lü et al. (11) showed that, after co-culturing endometrial epithelial and stromal cells with SMCs, the reconstructed tissue expressed *β3* integrin, heparin-binding epidermal growth factor-like growth factor, and HOXA-10. 

Our results showed no significant differences between the studied groups in the presence and absence of mouse embryos regarding the expression of genes related to implantation. It seems that epithelial-like cells derived from SUSD2^+^ stem cells and ECS in the presence of mouse embryo exhibit the same gene expression profile as that in the absence of an embryo. Thus so far, no evidence has been reported to evaluate the effects of embryos on the expression of genes related to implantation in cultured endometrial stem cells. In relation to this, Popovici et al. (39) have reported that co-culture of trophoblast with endometrial stromal cells reduces the expression of matrix metalloproteinase-11 and increases the expression of IL-1 receptors in these cells. It has been suggested that the difference in the species sources of embryo and cultured cells (human endometrial cells and mouse embryos) in our study can affect the expression pattern profile of genes related to implantation and/or the expression of these genes may be time-dependent. Considering that implantation has a wide genomic profile, gene expression analyses in this study were not timed according to their *in vivo* time of expression. Moreover, possibly during the expansion of SUSD2^+^ cells in culture, they undergo some changes depending on cell density, cell-cell contact, and Notch signalling (40). In the present study, the endometrial tissue samples were collected from a population between 25 and 40 years of age. It should be mentioned that the age of human samples as a source of the endometrial cells might affect embryo implantation and the expressions of genes related to implantation. Nevertheless, due to a limited sample size and some limitations to prepare more human tissue in this study, this should be considered in further investigations. 

## Conclusion

This study showed that SUSD2^+^ cells during co-culture with SMCs can interact with mouse embryos. These co-cultured cells have the potential to be used as an implantation model.

## Supplementary PDF


