# Supplementary material for: In Vitro Implantation Model Using Human Endometrial SUSD2+
Source: Cell J. 2021 May 26;23(2):154–63. doi: 10.22074/cellj.2021.6979 (PMC8181319; doi:10.22074/cellj.2021.6979)
Supplement: Supplementary file 1 [file Cell-J-23-154-s01.pdf]

CELL JOURNAL (Yakhteh)

www.celljournal.org

Supplementary Information for

In Vitro Implantation Model Using Human Endometrial SUSD2+ Mesenchymal Stem Cells and Myometrial Smooth Muscle Cells

Marzieh Rahimipour, Ph.D.[1], Mina Jafarabadi, M.D.[2], Mojdeh Salehnia, Ph.D.[1]*

1. Department of Anatomy, Faculty of Medical Sciences, Tarbiat Modares University, Tehran, Iran
2. Reproductive Health Research Centre, Tehran University of Medical Sciences, Tehran, Iran

***Corresponding Address: P.O.Box: 14115-111, Department of Anatomy, Faculty of Medical Sciences, Tarbiat Modares University, Tehran, Iran
Email: salehnim@modares.ac.ir***

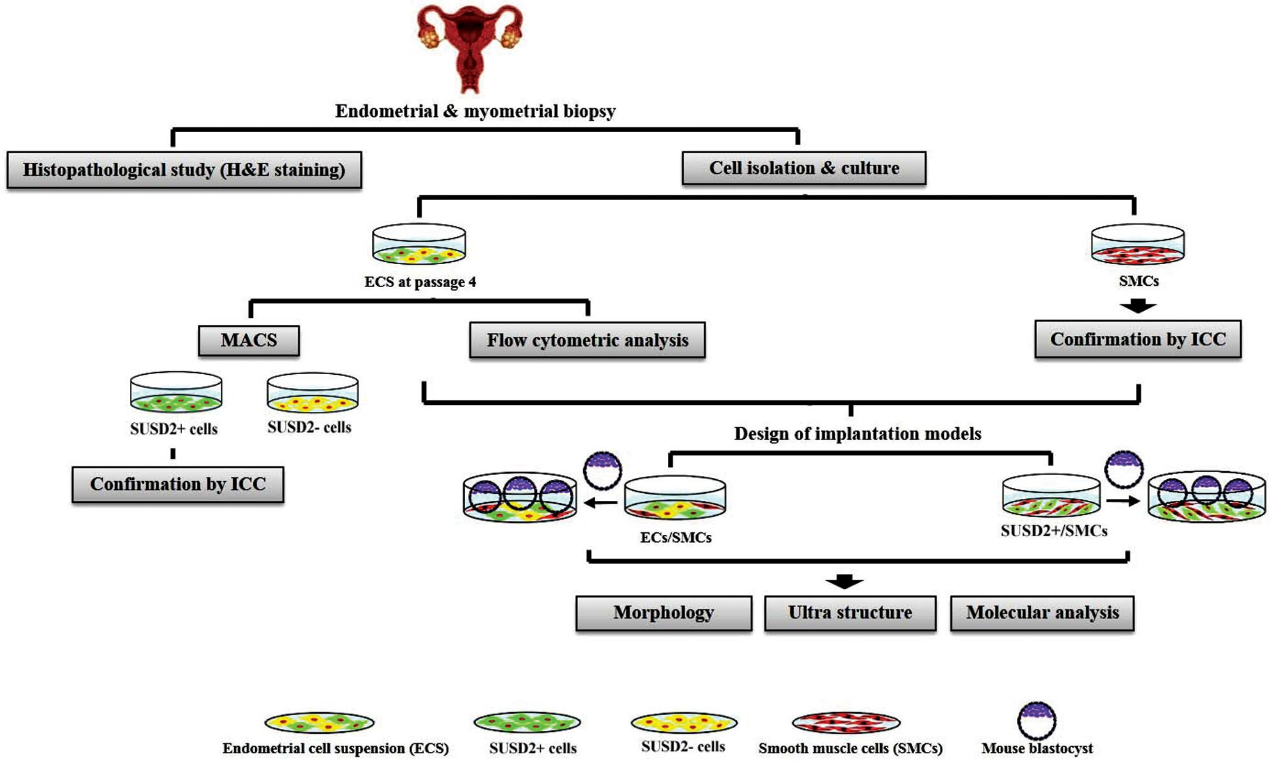

Fig.S1: Schematic diagram of the experimental design.

Table S1: Characteristics of the human samples

Samples (n)	Age (Y)	Menstrual cycle stage	Tissue samples	BMI	Drug (hormones)
1	40	Proliferative	Endometrium	26	No
2	40	Proliferative	Endometrium	21	No
3	25	Proliferative	Endometrium	24	No
4	39	Proliferative	Endometrium	20	No
5	31	Proliferative	Endometrium	19	No
6	40	Proliferative	Endometrium	21	No
7	30	Proliferative	Endometrium	23	No
8	40	Proliferative	Endometrium	23	No
9	33	Proliferative	Endometrium	20	No
10	40	Proliferative	Endometrium	24	No
11	29	Proliferative	Myometrium	20	No
12	31	Proliferative	Myometrium	22	No
13	40	Proliferative	Myometrium	22	No
14	35	Proliferative	Myometrium	24	No
15	40	Proliferative	Myometrium	23	No
16	40	Proliferative	Myometrium	25	No
17	38	Proliferative	Myometrium	21	No
18	40	Proliferative	Myometrium	22	No
19	29	Proliferative	Myometrium	22	No
20	40	Proliferative	Myometrium	23	No

BMI; Body mass index.
